# Cushing’s Syndrome Presenting with Depression as the Initial Manifestation: A Case Report and Literature Review

**DOI:** 10.2174/0118715303397972250811050815

**Published:** 2025-08-13

**Authors:** Xiuli Xu, Baoqiang Guo, Xuexiu Chi

**Affiliations:** 1 Electrocardiogram Room (EEG Room), The Second People’s Hospital of Liaocheng City, Liaocheng, 252600, Shandong Province, China;; 2 Department of Endocrinology, The Second People’s Hospital of Liaocheng City, Liaocheng, 252600, Shandong Province, China

**Keywords:** Cushing’s syndrome, depression, adrenocortical adenoma, delayed treatment, case report, dexamethasone suppression tests

## Abstract

**Background:**

In approximately 12% of Cushing’s syndrome (CS) cases, psychiatric symptoms, such as depression and anxiety, are the initial manifestation, obscuring the diagnosis and leading to delayed treatment.

**Case Summary:**

This case report describes a patient whose initial symptom of CS was low mood. Eighteen months later, typical clinical features of CS emerged, and the diagnosis was confirmed through dexamethasone suppression tests and adrenal computed tomography. This report also reviews relevant literature, analyzing the clinical features, pathogenesis, treatment, and prognosis of CS.

**Conclusion:**

In patients presenting with low mood or depression but no typical CS features, clinicians should consider CS as a potential underlying cause. Endocrinologists should be vigilant when evaluating patients with mental disorders. Timely diagnostic evaluations can lead to earlier diagnosis and treatment, ultimately improving prognosis.

## 1 INTRODUCTION

Cushing’s syndrome (CS) is a clinical syndrome characterized by excessive secretion of glucocorticoids by the adrenal glands due to various causes. The estimated annual incidence of endogenous CS is 2-8 cases per million people [1], and the condition predominantly affects women aged 30-40 years at a ratio of 3-4:1 compared to men [2]. According to the underlying causes, the condition can be divided into adrenocorticotropic hormone (ACTH)-dependent and adrenocorticotropic hormone (ACTH)-independent CS [3, 4]. Approximately 80%-85% of CS cases are ACTH-dependent, whereas approximately 15%-20% are ACTH-independent [1, 5]. However, ACTH-independent CS accounts for the majority of cases in Japan and Taiwan [6]. Typical clinical features of CS include centripetal obesity, moon face, plethora, purple striae, hypertension, type 2 diabetes, and osteoporosis.

However, some patients exhibit atypical symptoms, such as emotional disturbances. Elevated cortisol levels in CS disrupt the limbic and cortical brain regions, leading to psychiatric symptoms that closely resemble primary depression and anxiety disorders [7, 8]. Presentation with only these early, nonspecific symptoms often delays diagnosis and treatment [9]. This case report examines the diagnosis and treatment of a patient with CS who initially presented with depression. A review of the relevant literature accompanies the case analysis, discussing the clinical characteristics, pathogenesis, treatment options, and prognosis of such cases of CS. The goal of this report is to aid clinicians in recognizing atypical presentations of CS, thereby reducing missed diagnoses and misdiagnoses.

## 2 CASE REPORT

A 23-year-old female student presented with an 18- month history of depressed mood and progressive weight gain, and a 4-month history of multiple purple striae. Her mood symptoms began without clear triggers and were accompanied by inattention, insomnia, sleep disturbance, fatigue, and reduced speech and movement. Her initial weight gain was mild. The patient denied any other abnormal behavior and at the time of presentation exhibited no typical Cushingoid features. Her score on the Hamilton Depression Rating Scale (HAMD) was 21, and her score on the Hamilton Anxiety Rating Scale (HAMA) was 18. Electrocardiogram findings were normal. She was diagnosed with depression and anxiety and treated with sertraline hydrochloride, but the response was limited.

Six months prior to her presentation in our department, the patient began to experience significant weight gain, particularly in the face and trunk, along with moon facies and pitting edema of the limbs. The endocrine evaluation revealed a cortisol level of 383.3 nmol/L (reference range: 172-497 nmol/L) and an ACTH level of 8.67 pg/mL (reference range: 7.20-63.30 pg/mL). Four months previously, she had developed widespread purple striae, hirsutism, acne, occasional palpitations, chest tightness, and irregular menstrual cycles, but no fever, hypertension, gastrointestinal symptoms, or muscle weakness. Abdominal computed tomography (CT) revealed a nodular low-density lesion in the right adrenal gland, measuring 2.1 cm × 1.8 cm × 2.2 cm. No treatment was initiated at that time.

On September 17^th^, 2022, the patient was admitted to the Department of Endocrinology at the Liaocheng Second People’s Hospital. An overnight low-dose dexamethasone suppression test revealed that cortisol levels were not suppressed at 8 am. At that time, the case was considered “right adrenal nodule, CS waiting to be diagnosed.” A timeline summarizing the progression of the patient’s condition is presented in Fig. (**1**).

The patient had a history of a left pubic fracture due to minor trauma 6 months prior, which was managed conservatively. She denied hypertension, any other chronic illness, or any medication use. Her menstrual history included irregular cycles without dysmenorrhea for the previous year. She was unmarried with no known fertility issues.

The physical examination on admission revealed a temperature of 36.7°C, a pulse of 104 beats/min, a respiratory rate of 20 breaths/min, a blood pressure of 141/98 mmHg, and a normal sensorium. She had a moon face, acne on her face and back, supraclavicular fat pads, and dorsocervical fat pads. Purple striae were present on her chest, abdomen, and limbs, with scattered ecchymosis on the limbs. No eyelid edema or thyroid swelling was observed. Breath sounds were clear bilaterally, with regular heart rhythm and no pathological murmurs. The abdomen was soft and non-tender, without hepatosplenomegaly or percussion tenderness. She had increased body hair and slight pitting edema in the limbs.

**Fig. (2):**
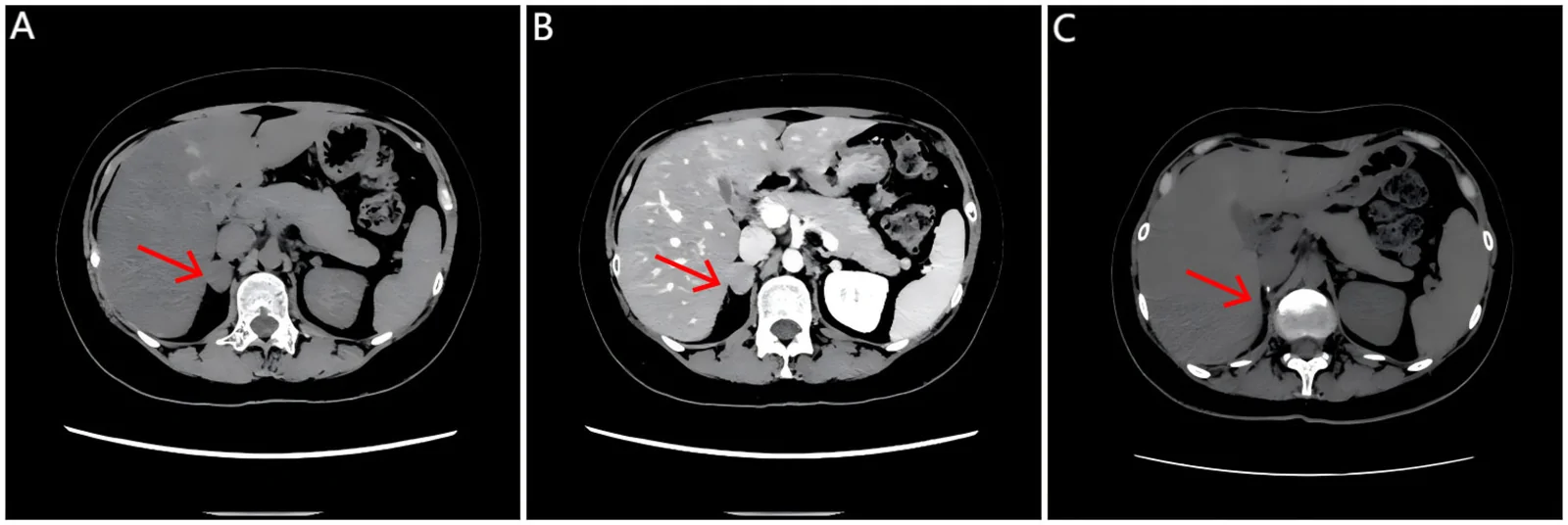
Adrenal CT imaging. (**A**) Preoperative plain CT scan showing a 20 mm × 18 mm nodule in the right adrenal gland (arrow). (**B**) Enhanced CT scan demonstrating significant enhancement of the right adrenal nodule (arrow), indicative of adrenal adenoma. (**C**) Postoperative plain CT scan (7 months after surgery) showing changes in the right adrenal gland consistent with surgical removal of the adenoma (arrow).

Upon admission, relevant examinations showed a HAMD score of 20 points and a HAMA score of 18 points. Her glycosylated hemoglobin level was 5.10%, and fasting glucose level was 5.36 mmol/L. Cortisol levels were elevated, with a rhythm of 434.2-384.6-370.6 nmol/L (8:00-16:00-0:00), and ACTH levels were low, with a rhythm of 2.35-2.03-4.26 pg/mL, respectively. Her 25-hydroxyvitamin D level was 14.77 ng/mL. Lipid profile analysis revealed triglyceride levels of 1.17 mmol/L, total cholesterol levels of 6.47 mmol/L, high-density lipoprotein (HDL) levels of 1.96 mmol/L, and low-density lipoprotein (LDL) levels of 3.98 mmol/L. Thyroid function tests, as well as thyrotropin receptor antibody, thyroid peroxidase antibody, parathyroid hormone, and reproductive hormone levels, were normal. Test results for erythrocyte sedimentation rate, electrolyte levels, plasma osmotic pressure, urine osmotic pressure, growth hormone level, aldosterone level, renin level, 17-hydroxyprogesterone level, and dehydroepiandrosterone sulfate level were also normal. The 24-hour urinary-free cortisol level could not be measured due to institutional limitations. However, the results of the low-dose and high-dose dexamethasone suppression tests (DST) were positive, with post-overnight DST cortisol levels of 416.8 nmol/L, post-classic low-dose DST cortisol levels of 439.1 nmol/L, and post-high-dose DST cortisol levels of 368.6 nmol/L. Plain adrenal CT suggested a high likelihood of right adrenal adenoma (Fig. **2**). Magnetic resonance imaging findings of the sellar region were normal. Additional findings included moderate fatty liver on abdominal ultrasound, decreased bone mass (Z-value of 2.1) on bone mineral density (BMD) assessment, and multiple thyroid cysts (Thyroid Imaging Reporting and Data System Type 2) on thyroid ultrasound, with normal color Doppler ultrasound results.

**Fig. (3):**
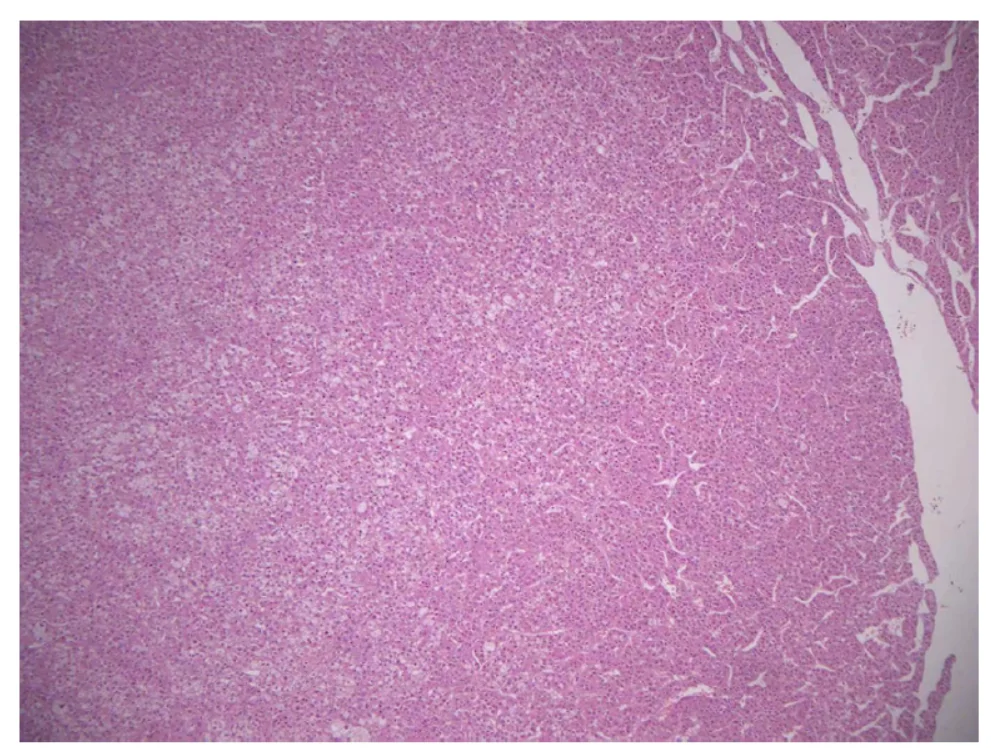
Histopathological examination of the resected right adrenal cortical adenoma specimen. Description: The specimen, measuring 2.5 cm × 2.3 cm × 2.3 cm, was fixed in formalin, embedded in paraffin, sectioned, and stained with hematoxylin and eosin (H&E). Microscopic evaluation at 100× magnification showed characteristic features of adrenocortical adenoma, including nests of uniform, eosinophilic tumor cells without significant atypia or mitotic activity.

**Fig. (4):**
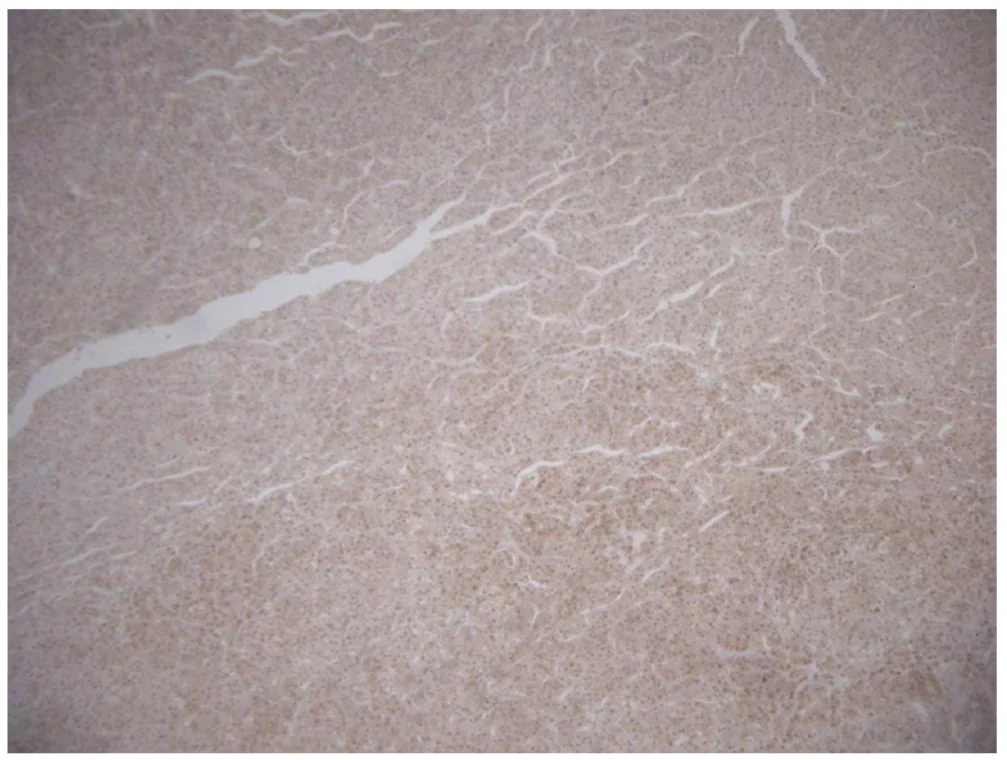
Immunohistochemical staining of the right ACA. Description: Tumor sections were subjected to immunohistochemistry using antibodies against calreticulin (CR), synaptophysin (Syn), and chromogranin A (CgA). The tumor showed strong cytoplasmic positivity for CR, supporting the diagnosis of a cortisol-producing adrenal adenoma. Focal positivity for Syn and CgA was also observed, reflecting partial neuroendocrine differentiation.

**Treatment and Prognosis:** The patient underwent laparoscopic right adrenalectomy in our hospital’s urology department on September 29^th^, 2022. Postoperative pathology confirmed right adrenocortical adenoma (ACA) measuring 2.5 cm × 2.3 cm × 2.3 cm (Fig. **3**). The tumor tissue was immunohistochemically positive for synaptophysin, indicating a neuroendocrine tumor, and chromogranin A (CgA), a marker of neuroendocrine cells. The normal adrenal cortex can express calreticulin (CR), which can distinguish epithelioid mesothelioma (positive) from adenocarcinoma (negative). CR positivity in the adrenal tumor tissue of this patient further supported ACA (Fig. **4**). The final diagnosis was right ACA causing CS. After surgery, the patient was started on prednisone acetate 15 mg as replacement therapy to protect against adrenal insufficiency while allowing time for recovery of the hypothalamic-pituitary-adrenal (HPA) axis. She experienced significant improvements, including gradual weight normalization, mood stabilization, resolution of purple striae and ecchymosis, and normalization of blood pressure, allowing her to discontinue oral medications. Her HAMD and HAMA scores decreased to 13 and 8 points, respectively. A follow-up on April 12^th^, 2023, showed no adenoma recurrence (Fig. **2**). Additionally, a significant increase in bone mass was observed on densitometry 6 months after surgery. Cortisol and ACTH levels gradually returned to normal during follow-up (Table **1**).

## 3 DISCUSSION

The patient in the presented case first experienced depression and a slight increase in weight and consulted a local psychiatric department. She was diagnosed with depression and anxiety, but her symptoms did not respond to antidepressant drugs. Based on her medical history and relevant examinations, 18 months later, she was ultimately diagnosed with CS, right adrenal cortical adenoma, depression and anxiety, abnormal blood lipid levels, secondary osteoporosis, and grade-1 hypertension.

Previous studies have reported that Cushing’s disease (CD) is responsible for 63.0% of CS cases, followed by ACA (20.9%), bilateral macronodular adrenal hyperplasia (6.2%), ectopic ACTH syndrome (5.9%), primary pigmented nodular adrenal cortical disease (1.8%), and adrenal cortical carcinoma (1.0%) [6, 10]. CS often presents with atypical early symptoms, including mental symptoms, such as depression and anxiety, which are the first symptoms of CS in as many as 12% of patients, whereas mania and psychosis are rare [10]. Severe depression affects 50%-80% of patients with active CD and has been found to occur at onset or even before diagnosis in up to 25% of cases [11]. Several case reports have described patients with CS who initially presented with psychiatric symptoms, including a manic episode [12], mental disorder and convulsions [13, 14], refractory depression [15], anxiety [16], and acute psychosis (Table **2**) [17]. However, relatively few CS cases with depression as the first manifestation have been reported in China. Our patient had early symptoms of depression and showed poor response to antidepressant therapy. Notably, elevated cortisol levels can lead to mental and neurocognitive disorders, and thus, clinicians should be vigilant for atypical symptoms of CS in the early stages and proactively inquire about psychiatric symptoms or utilize screening tools to identify potential issues. Even if endocrinologists cannot make a definitive psychiatric diagnosis, referral of patients to a psychiatrist for further evaluation and diagnosis can be beneficial [11].

The exact pathogenesis of cortisol-induced neuropsychiatric disorders remains unclear. However, research suggests that autonomous glucocorticoid production in CS is associated with a range of neuropsychiatric symptoms. Certain brain regions are particularly susceptible to prolonged exposure to glucocorticoids [18-20]. Cortisol exerts its effects through glucocorticoid receptors (GRs) and mineralocorticoid receptors (MRs), with its effects in the brain largely determined by the balance between GR and MR activation [10]. The central nervous system, specifically the hippocampus, amygdala, limbic system, and prefrontal cortex, is rich in GRs, making it especially susceptible to hypercortisolemia [11]. Several mechanisms have been proposed to explain the neuropsychiatric manifestations of CS. These include reduced cerebral glucose metabolism leading to brain atrophy, excessive release of excitatory amino acids, such as glutamate, causing dendritic atrophy in the hippocampus, decreased levels of neurotrophic factors like nerve growth factor and brain-derived neurotrophic factor (BDNF), and impaired neurogenesis in the dentate gyrus [20]. Imaging studies in CS patients have demonstrated reduced volume in the right hippocampus, a region essential for emotion regulation, memory, and learning, suggesting that hippocampal damage may underlie the cognitive deficits observed in these individuals [18]. Additionally, depression has been linked to both dysregulation of the HPA axis and exposure to chronic life stress. Persistent stress leads to elevation of cortisol levels, particularly affecting the hippocampus and decreasing BDNF expression. This contributes to neuronal loss in the CA3 region of the hippocampus, reduced serotonin secretion, and the development of mood and cognitive disorders [21].

The first-line treatment for CS is surgical resection of the tumor. Some patients require additional treatment, such as drug therapy, radiotherapy, or bilateral adrenalectomy [1], as summarized in Table **3**. Without effective treatment, CS can lead to various complications, including cardiovascular diseases (hypertension, atherosclerosis, impaired cardiac function), metabolic disorders (dyslipidemia, central obesity, diabetes), thrombotic diseases, bone diseases, and cognitive and neuropsychological disorders related to excess cortisol. Even after successful surgery, the risk of complications persists [22]. Postoperatively, patients may experience adrenal insufficiency, requiring glucocorticoid replacement therapy to slow the decline in cortisol levels and prevent associated mental disorders and cardiovascular events. The recovery time for adrenal insufficiency after surgery varies depending on the underlying cause of CS. Ectopic CS tends to have the shortest recovery time, followed by CD, while adrenal CS caused by a unilateral cortisol-producing adenoma typically has the longest recovery time [23]. Research has shown that elevated cortisol levels in patients with CS significantly affect their health-related quality of life. The duration of exposure is a critical factor, as longer exposure times can potentially lead to more severe and irreversible damage, ultimately affecting treatment outcomes and quality of life [24]. While antidepressants can increase BDNF expression and slow hippocampal atrophy, the effectiveness of these drugs may be reduced in patients with hypercortisolism. Additionally, antidepressants can cause side effects, such as liver function deterioration. Therefore, they are not typically recommended for symptomatic treatment in CS. Instead, treating the underlying cause of CS and normalizing cortisol levels can lead to gradual improvement in mental symptoms over time [11].

CS is a chronic condition requiring ongoing evaluation and monitoring for typical symptoms and complications. The patient in the presented case underwent laparoscopic surgery for removal of the adrenal adenoma and received prednisone treatment for approximately 6 months. After her cortisol and ACTH levels normalized, the medication was discontinued. The patient currently exhibits improved mental health and other symptoms, with normal levels of biochemical indicators. A suprarenal gland CT scan showed no signs of recurrence. Regular follow-up will be necessary to monitor her condition.

## CONCLUSION

Early CS can present with atypical symptoms, such as depression, leading to delayed diagnosis and treatment. Hypercortisolism can lead to mental and neurocognitive disorders, particularly depression and anxiety. Clinicians should be vigilant when evaluating patients with mental health disorders, considering the possibility of underlying organic diseases, such as CS. Screening tools can help identify these cases, enabling early diagnosis and treatment.

## STUDY LIMITATIONS

The study is based on a single patient, which limits the generalizability of the findings. Additionally, although the patient showed significant improvement, the follow-up duration (approximately 6 months post-surgery) is quite short for assessing long-term outcomes and potential recurrence. These limitations highlight areas for future research and case studies to build upon the findings presented in this report.

## Figures and Tables

**Fig. (1):**
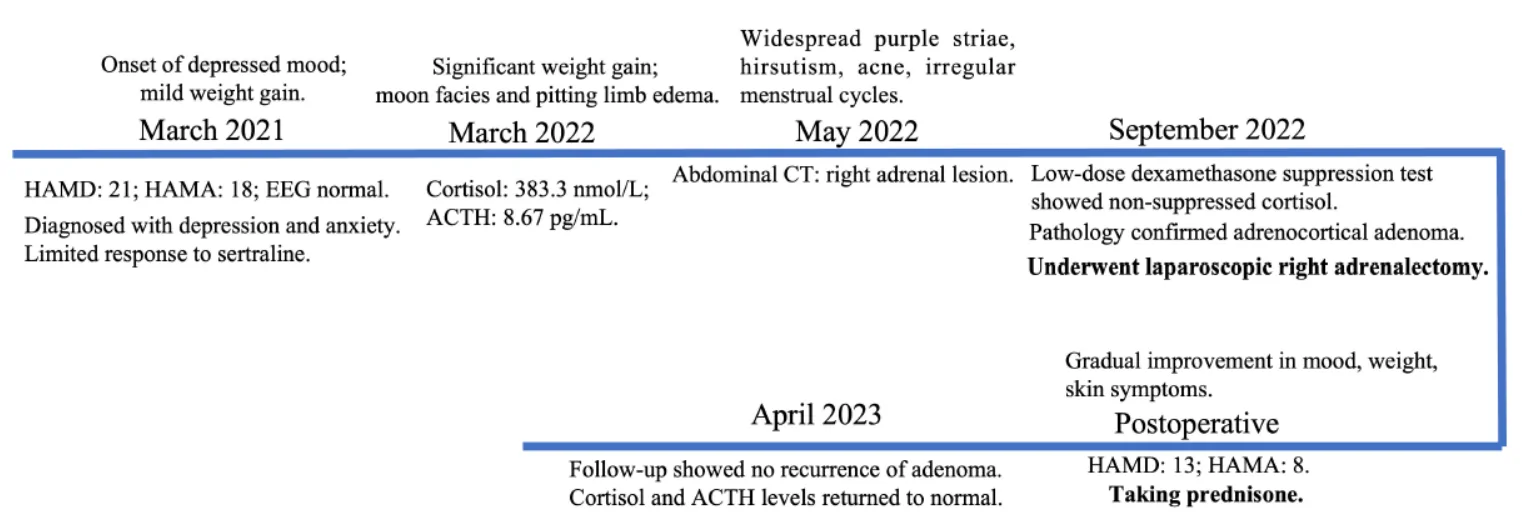
Timeline of the patient’s clinical course. **Abbreviations:** ACTH, adrenocorticotropic hormone; HAMD, Hamilton Depression Rating Scale; HAMA, Hamilton Anxiety Rating Scale; EEG, electroencephalogram.

**Table 1:** Changes in cortisol (8:00 h) and ACTH (8:00 h) levels during follow-up.

Detection Time	Cortisol (nmol/L)	ACTH (pg/mL)	Clinical Interpretation
**Before Surgery**			
2022-09-23	434.2	2.35	Typical Cushing’s pattern: Elevated cortisol with suppressed ACTH.
**After Surgery (date: 2022-09-29)**			
2022-10-15	8.26	5.12	Successful tumor removal resulted in a significant decrease in cortisol levels; ACTH began to recover from suppression.
2022-10-23	7.67	5.85	
2022-11-12	3.60	3.33	
2023-02-04	13.86	14.08	Gradual reactivation of the HPA axis, still on steroid replacement.
2023-03-02	111.1	37.9	Progressive **HPA axis recovery**; contralateral adrenal resuming cortisol production; external steroid has been tapered.
2023-04-12	257.4	51.61	
2023-07-15	265.3	36.57	

**Abbreviations:** ACTH: adrenocorticotropic hormone; HPA: hypothalamic-pituitary-adrenal.

**Table 2:** Published case reports of CS initially presenting with psychiatric symptoms.

Author(s)	Published Year	Diagnosis	Initial or Prominent Psychiatric Manifestation
Jia *et al*. [12]	2018	Ectopic ACTH syndrome	Manic episode
Zhao *et al*. [13]	2014	CS	Mental disorder and convulsions
Ma *et al*. [14]	2015	CS	Mental disorder as the first manifestation
Yin *et al*. [15]	2023	CS	Refractory depression and acute psychosis
Geng *et al*. [16]	2023	CS	Secondary anxiety syndrome
Daya *et al*. [17]	2022	CS	Schizophrenia-like psychosis

**Table 3:** Treatments for CS and their relevance to depression.

Treatment	Drug class / Approach	Mechanism of Action	Used for Depression	Notes
**Surgical Intervention**
	Transsphenoidal surgery	Removes ACTH-secreting pituitary adenoma	-	First-line treatment for CD.
	bilateral adrenalectomy	Removes both adrenal glands to eliminate the cortisol source	-	Last-resort. May worsen depression.
**Radiation Therapy**	Stereotactic radiosurgery	Destroys residual pituitary tumor	-	Used when surgery fails or is incomplete.
**Drug Therapy**				
Ketoconazole	Steroidogenesis inhibitor	Inhibits adrenal cortisol synthesis	-	
Metyrapone	Steroidogenesis inhibitor	Inhibits 11β-hydroxylase	-	
Mitotane	Adrenolytic agent	Cytotoxic to the adrenal cortex	-	
Osilodrostat	Steroidogenesis inhibitor	Inhibits 11β-hydroxylase	-	
Mifepristone	Glucocorticoid receptor antagonist	Blocks cortisol at the receptor level	Yes *(investigational)*	For psychotic depression and cortisol-related mood disorders.
Pasireotide	Somatostatin analog	Inhibits ACTH secretion *via* SSTR5 binding	-	
Cabergoline	Dopamine D2 receptor agonist	Suppresses ACTH release from the pituitary gland	Yes *(off-label)*	For mood improvement, especially in dopamine-related mood disorders.
SSRIs/SNRIs	Antidepressants	Increase serotonin/norepinephrine in the central nervous system	Yes *(standard)*	Not for CS per se, but for managing secondary depression.

**Abbreviations:** SSTR5: somatostatin receptor 5; SSRI: selective serotonin reuptake inhibitor; SNRI: serotonin-norepinephrine reuptake inhibitor.

## Data Availability

The raw data supporting the conclusions of this article will be made available upon request by the authors without any undue reservation.

## LIST OF ABBREVIATIONS

CS: Cushing’s Syndrome
HAMD: Hamilton Depression Rating Scale
HAMA: Hamilton Anxiety Rating Scale
BMD: Bone Mineral Density
TI-RADS: Thyroid Imaging Reporting and Data System
ACA: Adrenocortical Adenoma
HPA: Hypothalamic-Pituitary-Adrenal
BDNF: Brain-derived Neurotrophic Factor

## STANDARDS OF REPORTING

CARE guidelines were followed.
